# Vascular Plug-Assisted Retrograde Transvenous Obliteration of Portosystemic Shunts for Refractory Hepatic Encephalopathy: A Case Report

**DOI:** 10.1155/2014/391420

**Published:** 2014-03-05

**Authors:** Jonathan K. Park, Sung-Ki Cho, Stephen Kee, Edward W. Lee

**Affiliations:** ^1^Division of Interventional Radiology, Department of Radiology, UCLA Medical Center, David Geffen School of Medicine at UCLA, 757 Westwood Plaza, Suite 2125, Los Angeles, CA 90095-743730, USA; ^2^Department of Radiology, Samsung Medical Center, Sungkyunkwan University School of Medicine, Seoul 135-710, Republic of Korea

## Abstract

While balloon-assisted retrograde transvenous obliteration (BRTO) has been used for two decades in Asia for the management of gastric variceal bleeding, it is still an emerging therapy elsewhere. Given the shunt closure brought about by the procedure, BRTO has also been used for the management of portosystemic encephalopathy with promising results. Modified versions of BRTO have been developed, including plug-assisted retrograde transvenous obliteration (PARTO), where a vascular plug is deployed within a portosystemic shunt. To our knowledge, we present the first North American case of PARTO in the setting of a large splenorenal shunt for the management of portosystemic encephalopathy.

## 1. Introduction

Portosystemic shunts may result in chronic portosystemic encephalopathy, a debilitating condition that may be refractory to medical management [1, 2]. Balloon-occluded retrograde transvenous obliteration (BRTO) is an interventional therapy for the treatment of gastric fundal varices developing in the course of a portosystemic shunt vessel [3, 4]. Due to shunt closure by the procedure, BRTO has also been applied to treat portosystemic encephalopathy [4]. In terms of portosystemic shunt closure, a balloon catheter may have a limited role because balloon inflation *per se* provides temporary blockage of the shunt flow until forming the thrombotic shunt occlusion by the sclerosant. Instead, use of a vascular plug might provide more stable and permanent shunt occlusion.

Vascular plug-assisted retrograde transvenous obliteration (PARTO) has recently been utilized to treat gastric varices and hepatic encephalopathy in Korea [5]. While a recent series has been published reporting the use of portosystemic shunt embolization for hepatic encephalopathy [6], PARTO employs a distinct advantage in utilizing additional small particle embolization in addition to shunt closure. We present to our knowledge the first North American case of PARTO utilized in the setting of large splenorenal and splenocaval shunts secondary to chronic cirrhosis as a successful primary therapy for portosystemic encephalopathy, along with a technical description.

## 2. Case Report and Technique

A 55-year-old male patient with a history of hepatitis C cirrhosis diagnosed 15 years prior was referred to our clinic by hepatology for consultation on possible BRTO. Symptoms included ascites, a history of esophageal bleeding, and multiple episodes of hepatic hydrothorax, as well as stage II hepatic encephalopathy. The patient had a Model for End-Stage Liver Disease (MELD) score of 14 and Child-Pugh score C at the time of presentation to our clinic. His ascites was well managed medically, and his esophageal varices were stable following banding and treatment with propranolol.

In spite of these therapies, multiple prior hospital admissions were required due to recurrent abdominal pain and hepatic encephalopathy. Of note, the patient had never undergone prior therapy with transjugular intrahepatic portosystemic shunt (TIPS). Symptoms included forgetfulness, slow speech, and severe difficulty sleeping at night, despite medical therapy with Rifaximin and Lactulose. The most recent venous ammonia level prior to first PARTO therapy was 169 mcg/dL (normal < 45 mcg/dL). Physical examination was notable for sluggish speech, flattened affect, and asterixis. 

**Figure 1:**
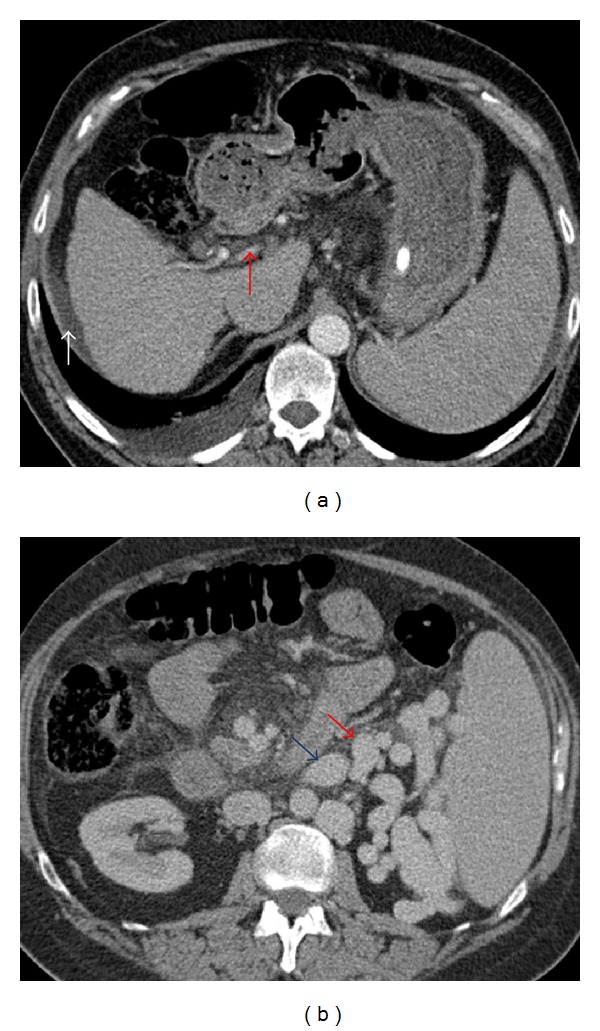
(a) Axial contrast-enhanced CT demonstrates diminutive portal vein (red arrow) and minimal perihepatic ascites (white arrow). (b) Axial CT demonstrates enlarged spleen with multiple perisplenic collateral vessels. A large splenorenal shunt (red arrow) is seen arising from a dilated left renal vein (blue arrow).

Contrast-enhanced computed tomography (CT) scan one week prior to clinic presentation demonstrated cirrhosis, minimal ascites, and a diminutive but patent portal vein (Figure 1(a)). There was marked splenomegaly with extensive collateral vessels medial to the spleen with a spontaneous splenorenal shunt (Figure 1(b)). Additionally noted was a portogonadal shunt, as well as a splenocaval shunt measuring approximately 12 mm in diameter. Due to the patient's well-controlled ascites and minimal esophageal varices, along with the presence of a large splenorenal shunt, he was thought to be an ideal candidate for BRTO or PARTO. The patient agreed to proceed with treatment and returned 5 days later.

**Figure 2:**
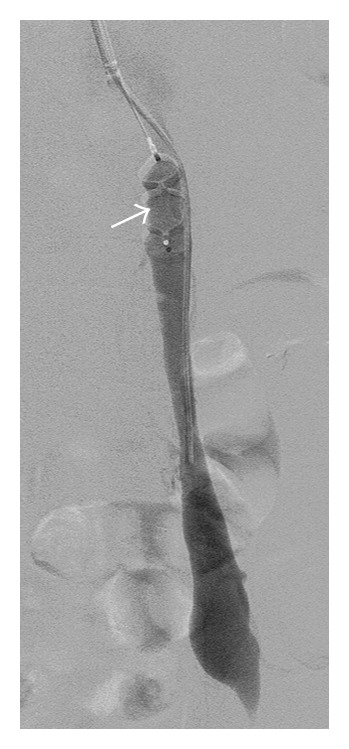
Venograms following selection of the left gonadal vein after Amplatzer plug deployment (arrow).

Following right internal jugular vein access with a 4-French micropuncture kit, a 14-French 30 cm vascular sheath was inserted into the right internal jugular vein. A 27 cm equalizer occlusion balloon catheter (Boston Scientific, Natick, Massachusetts) was inserted into the proximal left renal vein. With the balloon inflated, renal venography was performed to identify connections to the splenorenal and portogonadal shunts. Following removal of a balloon catheter, a portogonadal shunt was selected using a 4-French catheter and the catheter was exchanged for an 8-French Destination sheath (Terumo, Somerset, NJ). Before placing an Amplatzer vascular plug, a 4-Fr angled Glide catheter (Terumo, Somerset, NJ) was separately advanced through the sheath into the portogonadal shunt in order to check shunt flow after vascular plug deployment and to perform additional intervention for the remaining shunt flow. A 20 mm Amplatzer vascular plug (St. Jude Medical, Saint Paul, Minnesota) was deployed through the 8-Fr Destination sheath within the gonadal vein end of the shunt (Figure 2) and retrograde venography was performed through a 4-Fr angled Glide catheter in the portogonadal shunt beyond the deployed Amplatzer vascular plug. Additional embolization with gelfoam slurry (Gelfoam, New York, NY) was performed until the remaining shunt flow disappeared. 

**Figure 3:**
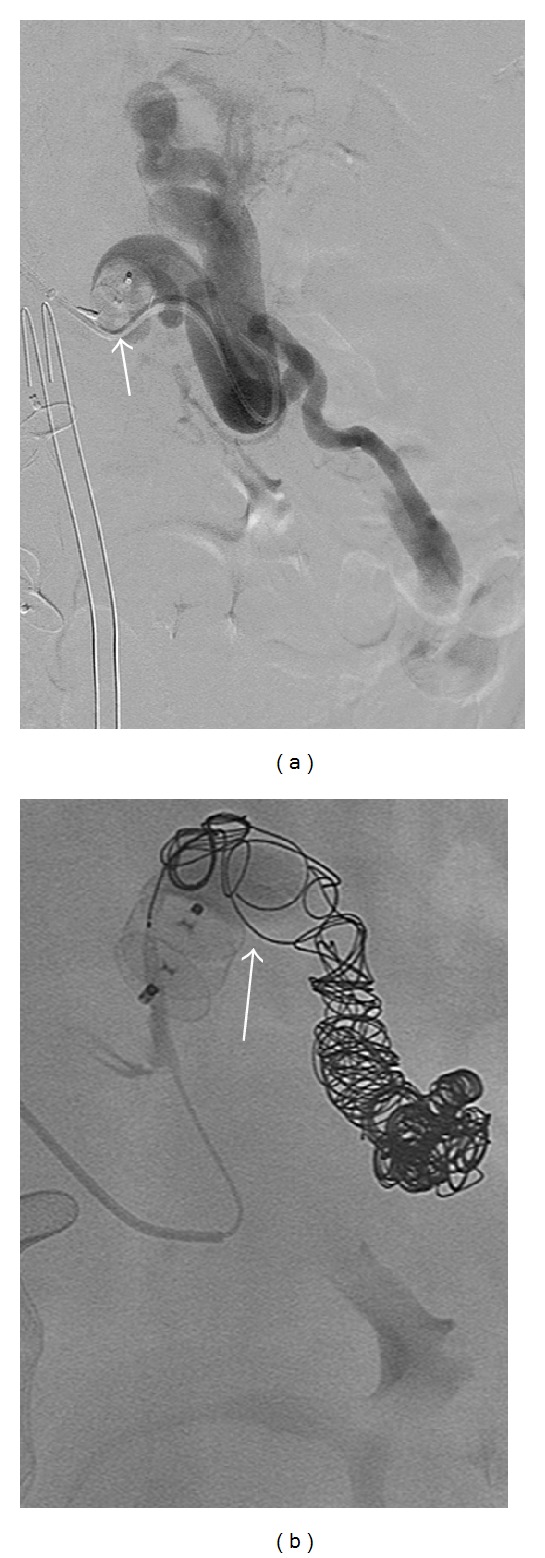
(a) Venogram after deployment of Amplatzer vascular plug (arrow) within splenorenal shunt. (b) Final image following placement of additional coils between initially placed coils and Amplatzer vascular plug.

For occlusion of the splenorenal shunt, the sheath was exchanged for a 10-French curved tip check flow sheath (Cook Medical, Bloomington, IN). The splenorenal shunt was selected with a 5-Fr Sos Omni catheter (AngioDynamics, Latham, NY) and the catheter was then exchanged to a 6-Fr 90 cm Destination sheath. With a 4-Fr angled Glide catheter separately kept in the more proximal part of the splenorenal shunt, a 14 mm Amplatzer vascular plug was deployed through the 6-Fr Destination sheath (Figure 3(a)). After advancing a Renegade STC microcatheter (Boston Scientific, Natick, Massachusetts) into the proximal part of the shunt, the splenorenal shunt was embolized with 0.035 inch Interlock microcoils (Boston Scientific, Natick, Massachusetts) and gelfoam slurry (Figure 3(b)). No immediate complications were observed.

**Figure 4:**
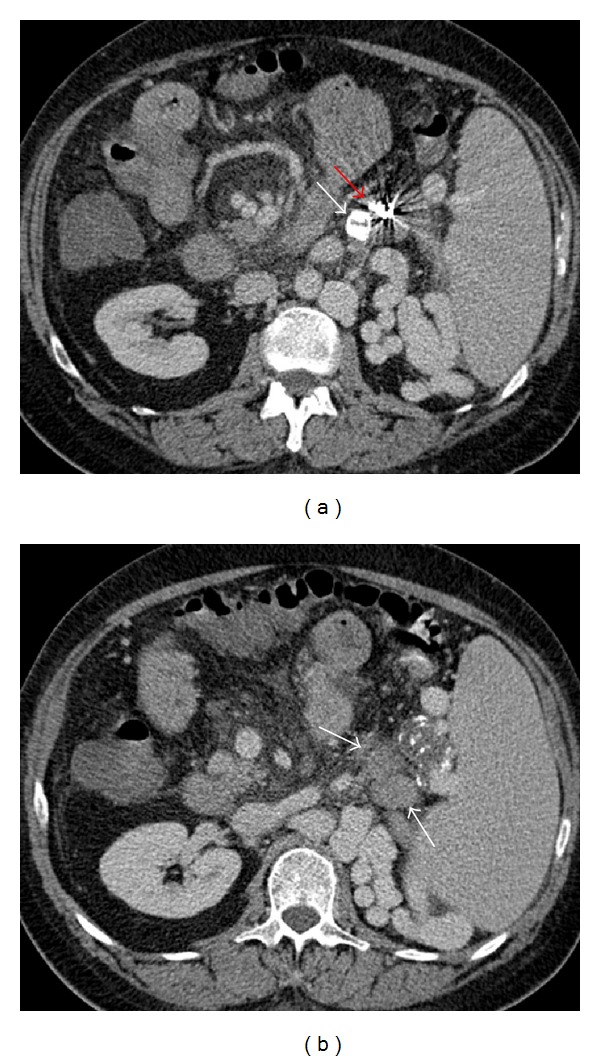
(a) Contrast-enhanced CT on postprocedure day 2 demonstrates Amplatzer vascular plug (white arrow) and coils (red arrow) within a splenorenal shunt. (b) White arrows demonstrate absence of flow distally in embolized splenorenal shunt.

A contrast-enhanced CT performed 2 days later demonstrated interval complete absence of perfusion of the gonadal and splenorenal shunts (Figure 4). The patient's ascites was stable. The postprocedure venous ammonia level was 68 mcg/dL. No other complications were visualized. Follow-up CT 3 months later showed persistent shunt occlusion, with stable smaller perisplenic collateral vessels.

The patient was seen 6 days following PARTO and was noted to have significant improvement in encephalopathy, which was stage II encephalopathy prior to PARTO. Mental status and cognitive function improved to the point of resolution as well. No further asterixis was demonstrated. The patient continued to demonstrate significant improvement at 4-month followup, and the only abnormality noted on physical examination was trace asterixis.

However, at 5-month followup, the patient was admitted to the hospital with recurrent right pleural effusion and symptoms of hepatic encephalopathy. Venous ammonia level was 134 mcg/dL, and a CT scan at this time demonstrated opacification of prominent perisplenic varices, thought to be secondary to a persistent splenocaval shunt that at this time was noted to measure 20 mm, as compared to 12 mm prior to first PARTO. 

**Figure 5:**
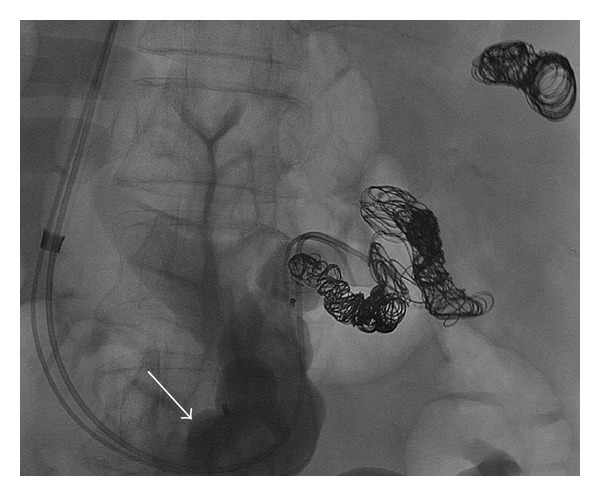
Catheter angiogram following coil and Gelfoam placement into the splenocaval shunt demonstrates draining collateral vessel (arrow) from the splenocaval shunt into the left renal vein, which was subsequently embolized with coils as well.

Repeat angiography was subsequently performed; although shunts originally closed with PARTO were still occluded, a persistent left splenocaval shunt was seen again, as well as collateral vessel extending from the shunt to the left renal vein (Figure 5). The large size of the splenocaval shunt precluded placement of vascular plug and embolization of both shunts with coils and Gelfoam was performed instead, with final angiogram demonstrating significant reduction in opacification of the patient's persistent perisplenic varices. At 1-day followup the patient demonstrated significant clinical improvement in encephalopathy with an immediate drop in venous ammonia level to 76 mcg/dL, and at 9-day followup his venous ammonia level decreased to 49 mcg/dL.

## 3. Discussion

In the case of portosystemic encephalopathy, the key factor in pathogenesis is shunting of portal venous blood into the systemic circulation. Thus shunt closure is a natural interventional target for relief of encephalopathy symptoms. Since its introduction, BRTO has been shown to be a suitable therapeutic option for patients with portosystemic encephalopathy due to various types of extrahepatic portosystemic shunts [7, 8].

The Amplatzer vascular plug has been used to safely treat various vascular conditions, including portosystemic shunts [6, 9]. Compared to a balloon catheter, the use of a vascular plug has several important benefits. First of all, the placement of the Amplatzer vascular plug on its own provides some embolic effect. In addition, PARTO utilizes gelfoam instead of sclerosing agents such as ethanolamine oleate utilized in BRTO, which are associated with complications including renal failure, pulmonary edema, and disseminated intravascular coagulation [5]. Balloon rupture during BRTO can also result in symptomatic pulmonary embolism, recurrent variceal bleeding, and treatment failure [10].

Clinical success in our case was likely brought about by reduction in shunting of undetoxified portal blood to the systemic circulation. In patients with gastric varices and hepatic encephalopathy treated by BRTO, the reported 1- and 3-year relapse-free survival rates are over 90% and 87%, respectively [11]. Given these excellent clinical outcomes, BRTO and related procedures such as PARTO have the potential to be first line therapies for the management of medically refractory portosystemic hepatic encephalopathy [12].

Amplatzer vascular plugs were initially placed both in portal-gonadal and splenorenal shunts in our case due to the presence of multiple portosystemic shunts. We believed that our patient was an ideal candidate for PARTO, given the finding of a large splenorenal shunt in the setting of medically refractory hepatic encephalopathy and elevated serum ammonia levels. The patient demonstrated angiographic and CT evidence of shunt occlusion, as well as initial clinical improvement of his previous grade II encephalopathy.

While the shunts originally occluded with PARTO remained closed, due to the preexisting presence of extensive collateral vessels, the patient experienced a recurrence of encephalopathy 5 months following the initial procedure. It was thought that this was secondary to a preexisting splenocaval shunt seen on initial CT examination, and on 5-month followup scan was noted to measure 20 mm (67% increase from initial 12 mm shunt size). On first presentation to us, there were multiple extensive collateral vessels, and this shunt was not embolized on initial procedure. Similar findings have also been described previously, specifically that portal hypertensive changes can be aggravated following transvenous obliteration, due to redirection of blood flow away from gastrorenal shunts (worsening or new development of portosystemic collaterals was seen in 6 (32%) of 19 patients in that study) [13].

In conclusion, to our knowledge we present the first case of PARTO in North America for the management of portosystemic encephalopathy. While BRTO has been performed for 2 decades in Korea and Japan, it is a relatively new addition to treatment options in North America and Europe. As this and other promising procedures such as PARTO continue to be performed internationally, the cumulative experience should result in increased data on long-term results, as well as continued development and refinement of techniques.
